# Dataset of quantitative spectral EEG of different stages of kindling acquisition in rats

**DOI:** 10.1016/j.dib.2017.11.045

**Published:** 2017-11-16

**Authors:** Mostafa Jalilifar, Ali Yadollahpour

**Affiliations:** Department of Medical Physics, School of Medicine, Ahvaz Jundishapur University of Medical Sciences, Ahvaz, Iran

**Keywords:** Kindling stage, Quantitative assessment, Spectral power, Rat

## Abstract

The data represented here are in relation with the manuscript "Quantitative assessments of extracellular EEG to classify specific features of main phases of seizure acquisition based on kindling model in Rat" (Jalilifar et al., 2017) [1] which quantitatively classified different main stages of the kindling process based on their electrophysiological characteristics using EEG signal processing. The data in the graphical form reported the contribution of different sub bands of EEG in different stages of kindling- induced epileptogenesis. Only EEG signals related to stages 1–2 (initial seizure stages (ISSs)), 3 (localized seizure stage (LSS)), and 4–5 (generalized seizure stages (GSSs) were transferred into frequency function by Fast Fourier Transform (FFT) and their power spectrum and power of each sub bands including delta (1–4 Hz), Theta (4–8 Hz), alpha (8–12 Hz), beta (12–28 Hz), gamma (28–40 Hz) were calculated with MATLAB 2013b. Accordingly, all results were obtained quantitatively which can contribute to reduce the errors in the behavioral assessments.

**Specifications Table**

**Table:** 

Subject area	Neuroscience
More specific subject area	Computational neuroscience
Type of data	Table, graph
How data was acquired	EEG signal processing using MATLAB 2013b.
Data format	Raw, analyzed
Experimental factors	Adult male rats (*n*=16) weighing 200±10 g were maintained in individual cages with an ambient temperature (25±2 °C) and 12-h dark: 12-h light cycle and after surgery underwent rapid Amygdala kindling model. The recorded field potentials during kindling progression were recorded and spectral analyses were performed to quantitatively assess the main three phases of epileptogenesis.
Experimental features	Computational analysis: EEG signals were analyzed using FFT
Data source location	Imam Khomeini Hospital, Ahvaz, Iran 31°18′11.5″N 48°44′41.9″E
Data accessibility	All of the data presented in this study are accessible within this article

**Value of the data**

• The data show the differences between the EEG signals of the kindling and control animals.
• The reported data can be used to develop seizure prediction model for temporal lobe epilepsy using EEG signals.
• Our data can contribute to explore the patterns of the kindling- induced epileptogenesis progression which can be useful to develop antiepileptic approach.

## 1 Data

The data of this study were collected from an animal in vivo study aiming at quantitative assessment of epileptogenesis in a rapid kindling model in rats [1]. Considering the unique features of EEG for seizure prediction [2], these data present the raw data of spectral analyses of the field potentials recorded during the progression of Amygdala kindling in rats to determine the quantitative features of main phases of kindling acquisition. In this paper, stages 1 and 2 of kindling were considered initial seizure stages (ISSs), stage 3 as localized seizure stage (LSS), and stages 4 and 5 as generalized seizure stages (GSSs). Table 1, Table 2, Table 3 present the spectral powers of different sub bands of EEGs in ISSs, LSSs, and GSSs of the kindling process, respectively. Moreover, Table 4 presents percentage of different sub bands power in the control group.

**Table 1:** Contribution of different sub bands power in ISSs. We reported the mean value for each rat.

	Rats	Delta	Theta	Alpha	Beta	Gamma
Kindle ISSs	Rat 1	0.64	0.29	0.03	0.03	0.01
	Rat 2	0.34	0.37	0.14	0.12	0.03
	Rat 3	0.52	0.26	0.1	0.1	0.02
	Rat 4	0.55	0.28	0.09	0.07	0.02
	Rat 5	0.4	0.43	0.08	0.06	0.01
	Rat 6	0.73	0.22	0.03	0.02	0.0047
	Rat 7	0.46	0.31	0.1	0.1	0.03
	Rat 8	0.56	0.28	0.07	0.08	0.01
	Rat 9	0.58	0.29	0.06	0.06	0.01
	Rat 10	0.45	0.33	0.09	0.1	0.03

**Table 2:** The percentage of different frequencies in LSSs. We reported the mean value for each rat.

	Rats	Delta	Theta	Alpha	Beta	Gamma
Kindle LSSs	Rat 1	0.4	0.31	0.09	0.16	0.03
	Rat 2	0.32	0.34	0.12	0.18	0.03
	Rat 3	0.23	0.25	0.17	0.32	0.03
	Rat 4	0.44	0.31	0.12	0.1	0.01
	Rat 5	0.32	0.36	0.09	0.19	0.04
	Rat 6	0.52	0.28	0.07	0.12	0.02
	Rat 7	0.22	0.37	0.2	0.18	0.03
	Rat 8	0.59	0.3	0.05	0.06	0.01
	Rat 9	0.47	0.31	0.09	0.11	0.02
	Rat 10	0.57	0.26	0.08	0.08	0.01

**Table 3:** contribution of different sub bands power in GSSs. We reported the mean value for each rat.

	Rats	Delta	Theta	Alpha	Beta	Gamma
Kindle GSSs	Rat 1	0.36	0.3	0.15	0.16	0.03
	Rat 2	0.35	0.32	0.13	0.18	0.03
	Rat 3	0.23	0.35	0.17	0.23	0.02
	Rat 4	0.27	0.29	0.2	0.23	0.02
	Rat 5	0.5	0.27	0.07	0.13	0.02
	Rat 6	0.61	0.28	0.05	0.06	0.01
	Rat 7	0.4	0.28	0.14	0.17	0.02
	Rat 8	0.56	0.34	0.05	0.04	0.01
	Rat 9	0.42	0.37	0.1	0.1	0.02
	Rat 10	0.59	0.27	0.69	0.06	0.01

**Table 4:** Contribution of different sub bands power in sham group. We reported the mean value for each rat.

	Rats	Delta	Theta	Alpha	Beta	Gamma
Sham	Rat 1	0.21	0.31	0.13	0.27	0.07
	Rat 2	0.24	0.26	0.15	0.25	0.09
	Rat 3	0.28	0.32	0.13	0.2	0.07
	Rat 4	0.3	0.29	0.12	0.2	0.06
	Rat 5	0.24	0.28	0.14	0.27	0.07
	Rat 6	0.32	0.26	0.14	0.21	0.07
	Rat 7	0.24	0.3	0.16	0.25	0.06

## 2 Experimental design

### 2.1 Materials and methods

Adult male rats weighing 200±10 g were housed individually under standard conditions (an ambient temperature (25±2 °C) and 12-h light: 12-h dark: 12-h light cycle).

**Fig. 1:**
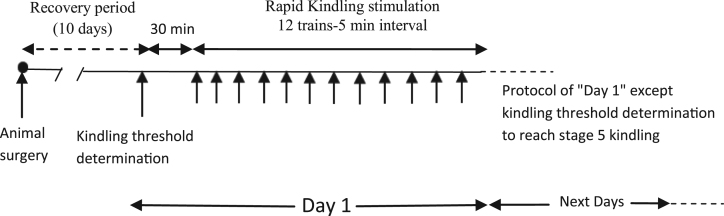
Schematic of the stimulation protocol.

Rats were randomly divided into two groups (ten for the kindle group and 6 for sham) and anesthetized under intraperitoneal injection of ketamine (100 mg/kg) and Xylazine (10 mg/kg) mixture [3]. One tripolar stainless steel electrode (a bipolar for stimulating and a monopole for recording EEG signal) was implanted in amygdala using Paxinos and Waston atlas coordinates: for amygdala targeting, anteroposterior: -2.5 mm; lateral: 4.8 mm; vertical: 7.2 and 0.2 mm below the skull [4]. Three holes were drilled, one for positioning a monopolar electrode attached to a screw which was located near the frontal lobe as ground and reference, the two for anchor screws. Electrodes and screws were fixed using acrylic dental cement and attached to a socket. The protocol of this study was approved by local ethics committee of Ahvaz Jundishapur University of Medical Sciences (Code: U-94147) that was in complete compliance to the guide for the care and use of laboratory animals by the National Institutes of Health (National Institutes of Health publication No. 86-23). Following a 10-day recovery period after surgery, the threshold intensity was determined using a 3 s of monophasic square wave of 50 Hz initially applied at 30 µA and it was increased in step of 15 µA at 15 min intervals until emerging at least 6 s of afterdischarges (ADs). All rats in the kindle group were subjected to daily stimulation using a 3 s train of 50 Hz monophasic pulses of 1ms duration with threshold intensity which were applied 12 times daily with 5 min intervals [5], whereas sham animals only experienced stimulation condition and received placebo stimulation (Fig. 1). Therefore, the EEG of sham animals can be considered as a baseline. Behavioral development of kindling acquisition was scored according to Racine stages [6]. This process was continued until emerging stage 5 of kindling. EEG signals recorded from the implanted electrode in the amygdala and monitored with electro module system (Tehran, Iran) which was connected to computer using e-probe software. During kindling acquisition, we could save the starting and ending time of each stage of kindling as a text file (an event file) which can be considered in extracting each stage. Data were digitized at a sampling rate of 10 KHz. Moreover, the electro module automatically applied a filter on 50 Hz frequency to remove DC effect from the signals. Recorded EEG signals were saved as binary files. These binary files were then imported into EEGLAB software for pre-processing stage. Moreover, a band-pass filter between 0.5–60 was applied to remove the effect of other frequencies. In the EEGLAB, we separated the EEG signals of each stage and the obtained signals were saved as dataset files which can be imported into MATLAB. These signals were then transferred into frequency domain by Fast Fourier Transform (FFT) and MATLAB 2013b was used to calculate their power spectrum and power of each sub bands including delta (1–4 Hz), Theta (4–8 Hz), alpha (8–12 Hz), beta (12–28 Hz), and gamma (28–40 Hz).
